# Point-of-care pancreatic stone protein measurement in critically ill COVID-19 patients

**DOI:** 10.1186/s12871-023-02187-w

**Published:** 2023-06-30

**Authors:** Gabriele Melegari, Enrico Giuliani, Giulia Di Pietro, Francesco Alberti, Mattia Campitiello, Elisabetta Bertellini, Consortium, Alessandra Rosa, Arianna Pioda, Paolo Battaglia, Maddalena Quarto, Enrico Ferri, Alberto Barbieri

**Affiliations:** 1 Department of Anaesthesia and Intensive Care, Azienda Ospedaliero Universitaria Di Modena, Modena, Italy; 2 Neuron Guard Ltd, Cambridge, UK; 3 School of Anaesthesia and Intensive Care, University of Modena and Reggio Emilia, Modena, Italy

**Keywords:** Pancreatic Stone Protein, Mortality predictor, COVID-19-ICU patients

## Abstract

**Introduction:**

Pancreatic stone protein (PSP) is a novel biomarker that is reported to be increased in pneumonia and acute conditions. The primary aim of this study was to prospectively study plasma levels of PSP in a COVID-19 intensive care unit (ICU) population to determine how well PSP performed as a marker of mortality in comparison to other plasma biomarkers, such as C reactive protein (CRP) and procalcitonin (PCT).

**Methods:**

We collected clinical data and blood samples from COVID-19 ICU patients at the time of admission (T0), 72 h later (T1), five days later (T2), and finally, seven days later. The PSP plasma level was measured with a point-of-care system; PCT and CRP levels were measured simultaneously with laboratory tests. The inclusion criteria were being a critical COVID-19 ICU patient requiring ventilatory mechanical assistance.

**Results:**

We enrolled 21 patients and evaluated 80 blood samples; we found an increase in PSP plasma levels according to mixed model analysis over time (*p* < 0.001), with higher levels found in the nonsurvivor population (*p* < 0.001). Plasma PSP levels achieved a statistically significant result in terms of the AUROC, with a value higher than 0.7 at T0, T1, T2, and T3. The overall AUROC of PSP was 0.8271 (CI (0.73–0.93), *p* < 0.001). These results were not observed for CRP and PCT.

**Conclusion:**

These first results suggest the potential advantages of monitoring PSP plasma levels through point-of-care technology, which could be useful in the absence of a specific COVID-19 biomarker. Additional data are needed to confirm these results.

**Graphical Abstract:**

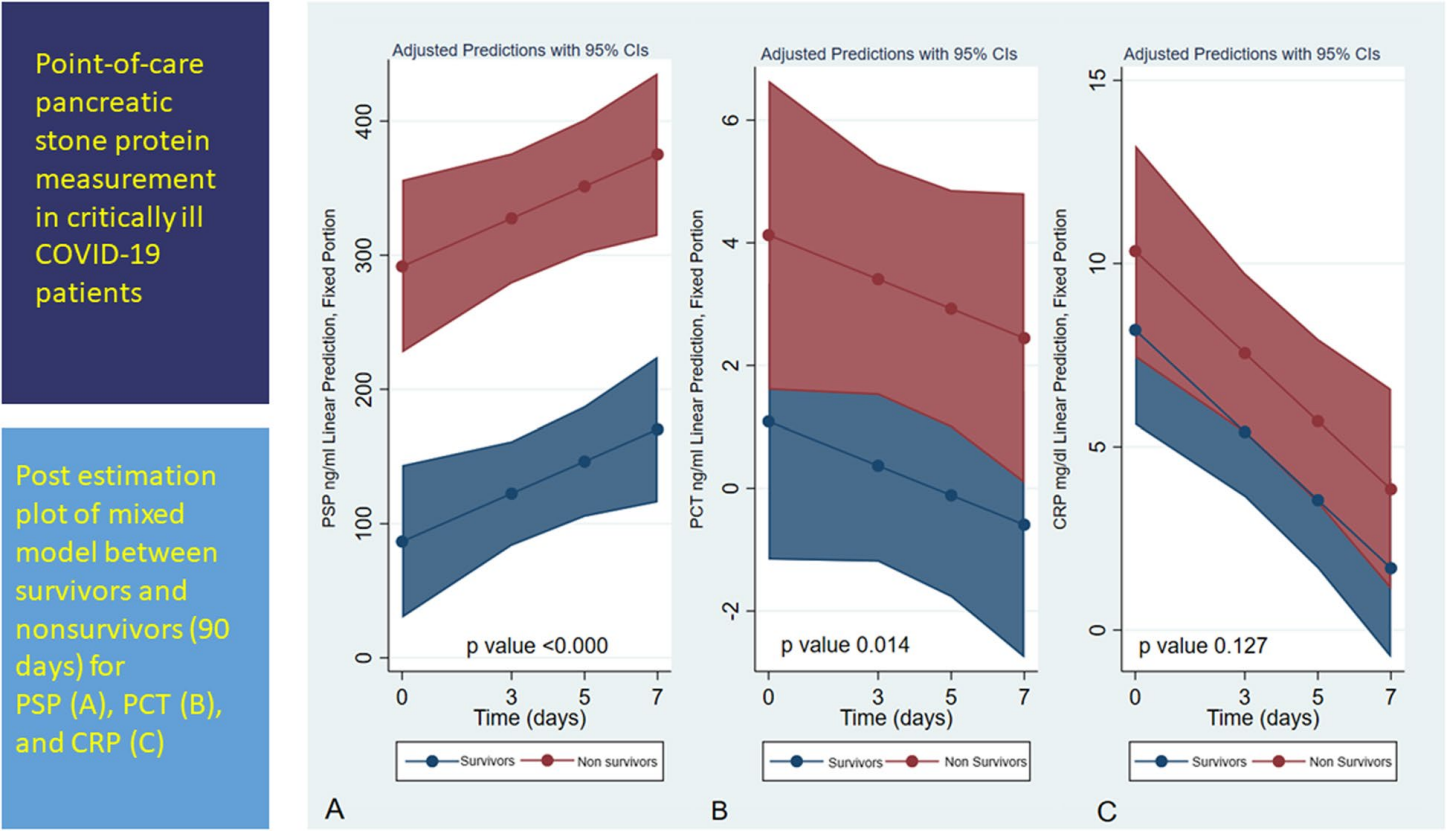

## Introduction

Pancreatic stone protein (PSP) is a novel biomarker that is reported to be increased in pneumonia and acute conditions, and PSP appears to be superior to procalcitonin (PCT) in discriminating among patients with infection, infection with sepsis, and no infection [1]. Recently, Van Singer et al., [2] described the potential advantages of measuring plasma PSP levels in critically ill COVID-19 patients due to the abnormal endothelial activation and inflammatory cytokine cascade that can be observed in COVID-19. Based on these assumptions, the lack of demonstrated clinical biomarkers in coronavirus disease progression requires researchers to further investigate the prognostic role of PSP in critically ill COVID-19 patients [3, 4]. The measurement of PSP in critical settings has found its application, especially in case of sepsis or in combination with other biomarkers [5]. Recently Klein et al. described the potential prognostic role of PSP in burns victims, showing the ability to differentiate between septic and non-septic patients during acute burn care [6]. The serial measurement of this protein over time has shown a prognostic value more than PCT and C-reactive protein (CRP) according to Pugin et al. [7]. According to Lagadinou et al., the measurement of PSP in hyper-inflammation, such as COVID-19 inflammation, could accurately identify patients requiring prolonged hospitalization [8].

## Aim of the study

In a proof-of-concept analysis, we measured PSP, PCT, CRP plasma levels in multiple blood samples, and we analysed these parameters as predictors of long term-care mortality (90 days). The primary endpoint was to study PSP plasma levels prospectively in a COVID-19 intensive care unit (ICU) population to define how well it performed as a marker of mortality in comparison to other plasma biomarkers such as CRP and PCT.

## Methods

Ethical approval was granted by the Ethics Committee of our health system (Azienda Ospedaliero Universitaria di Modena, reference number 784/2021) and the study was carried out in accordance with relevant guidelines and regulations in the Declaration of Helsinki. Informed consent was obtained for all participants when it was possible, in case of impossibility it was waived according to Italian regulations. Patients admitted to a COVID-19 medical intensive care unit who received standard medical care as recommended in COVID-19 sepsis guidelines were screened from March 2021 to June 2021 [9–11] At the same time of blood sample collection, we retrospectively collected information on the mechanical ventilation type; systolic, diastolic and mean arterial pressure measured via a radial or femoral arterial catheter and recorded by the patient’s electronic monitor; urine output per day measured via a urinary catheter; vasopressor and inotropic support dosage; and daily prescribed therapy to describe the patient’s condition at baseline. Physiological variables and medications of interest, the Horowitz index or Sequential Organ Failure Assessment (SOFA) scores were also collected. Finally, patient survival was followed even after discharge from the ICU, and 90 days of mortality data was collected from an electronic register.

### Inclusion and exclusion criteria

Inclusion criteria were as follows: age over 18 years, arterial catheter in situ, respiratory failure with mechanical ventilation assistance, urinary catheter in situ, expected ICU length of stay > 24 h, and informed consent signed by patient or next-of-kin when possible, in line with Italian regulations. Exclusion criteria were as follows: patient or next-of-kin refusal or do-not-resuscitate disposals, previous admission to a COVID-19 ICU, and COVID-19-related hemorrhagic or ischemic stroke as the cause of admission to the ICU.

### Sample measurement

Patients were positioned lying flat for at least 1 h before the collection of a 4 mL blood sample from the arterial catheter into an ethylene diamine tetra-acetic acid tube (Vacutainer; Becton Dickinson, Franklin Lakes, NJ). PSP levels were measured in whole blood by point-of-care testing using nanofluidic technology (PSP fluorescent immunoassay on the abioSCOPE® IVD device, Abionic SA, Epalinges, Switzerland). PCT and CRP levels were measured upon admission through routine blood tests. Blood samples were drawn at the patient’s time of admission to the ICU, and in the morning (06:00–12:00), PSP, PCT, and CRP levels were measured at the time of admission (T0), 72 h later (T1), five days later (T2) and finally 7 days later (T3).

### Statistical analysis and sample size

We planned a priori analysis of variance (ANOVA), and mixed model test repeated measures correlation coefficients (Rrm) were used for within-patient comparisons, cons is the constant (Y intercept) of the model [12, 13]. A sample of 10 patients was necessary to have 95% power to detect f = 0.5 (medium size effect) for weekly variation of PSP with alpha = 0.05 (supplementary information, sample size file) [14]. Results were reported as the mean plus standard deviation (st. dev) and with the 95% confidence interval (CI). The area under the receiver-operator (AUROC) curves was used to test mortality predictions, and the p value (*p*) was considered significant if < 0.05. Analysis of variance, the Student T test, and the Wilcoxon rank sum test were also performed depending on type of variable. A probit model was applied for regression with a binary dependent variable. Pearson correlation analysis was performed for continuous variables. Postestimation margins were estimated to plot some results. All analyses were performed with STATA (version 16.0, Stata Corp, College Station, TX, USA) and G Power (Erdfelder, Faul, & Buchner, 1996) software was used to estimate sample size [15, 16].

## Results

### Patients

**Fig. 1:**
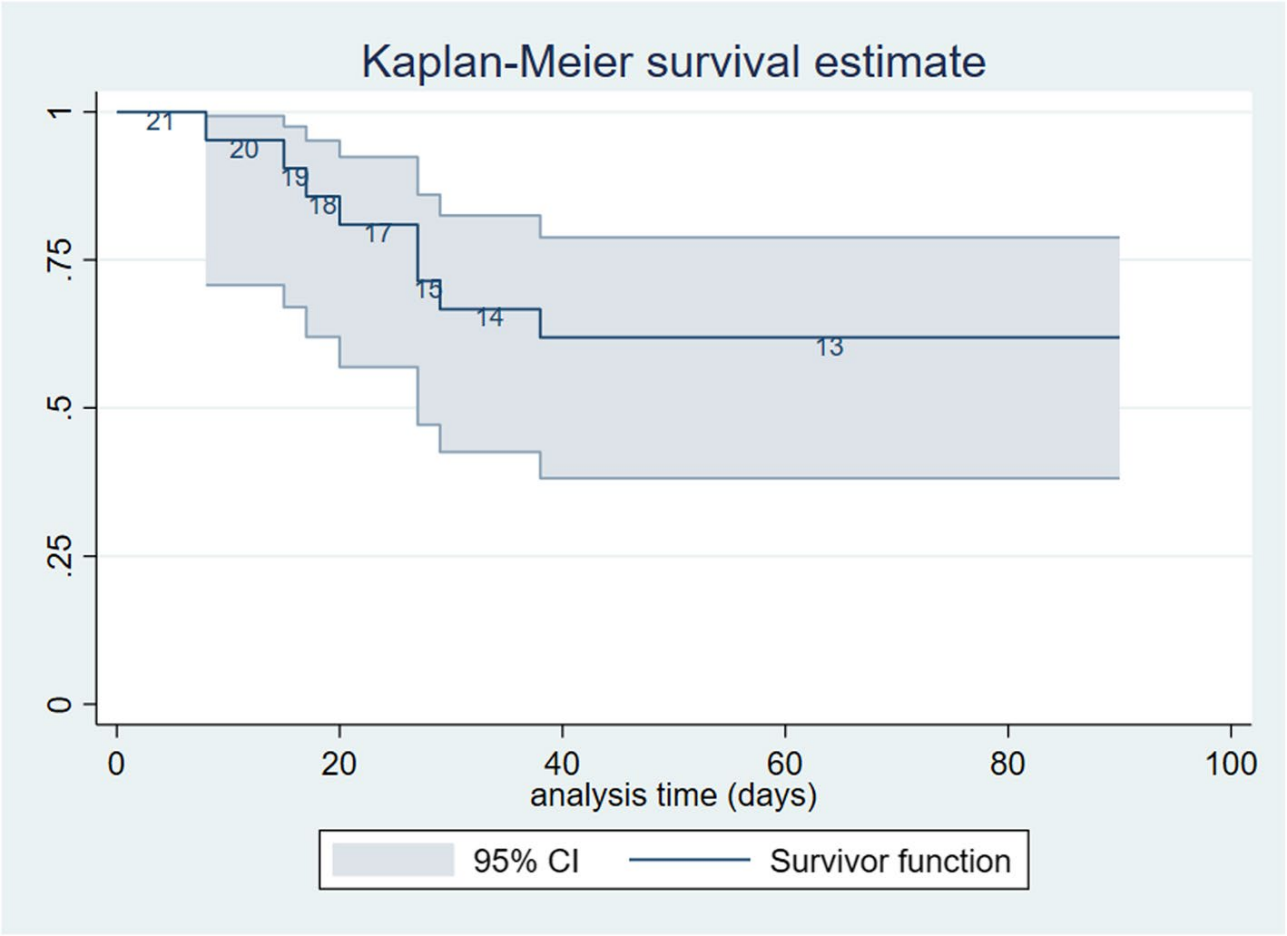
Shows the Kaplan Meier survival estimate of the study population and at-risk number in the Cox model

One hundred and nine patients were admitted to the COVID-19 ICU from March 1^st^ to June 1^st^ (90 days). According to the previously listed criteria, a total of twenty-one of these patients were enrolled in the study: 11 women (52.38%) and 10 males (47.62%). One hundred and thirty-eight blood samples were collected from the patients, and 84 blood samples were included in our analysis. We were unable to test plasma PSP in 2 patients, thus leaving 82 samples for analysis. Eight deaths occurred during the specified follow-up period resulting in a mortality rate of 38.10% over 90 days. The mean age of the population was 69.05 (CI 65.67 – 72.52) years, and no significant differences in age were observed between survivors and non-survivors. The most common comorbidities were hypertension, which was observed in 8 patients (38.10%); type 2 diabetes mellitus (DMII), 5 patients (23.81%); obesity, 3 patients (14.29%); chronic kidney failure (CKF), 2 patients (9.52%); and atrial fibrillation (9.52%) and chronic heart failure (9.52%). At the time of admission, there were no detectable differences between survivors and nonsurvivors regarding the Horowitz index or Sequential Organ Failure Assessment (SOFA) scores (Table 1 and Fig. 1).

**Table 1:** demographic conditions of the study population

Variable mean ± st.dev or %	Overall	Survivors (13 patients)	Non Survivors (8 patients)	*p* value
Age	69.04 ± 7.63	67.69 ± 8.54	71.25 ± 5.70	0.259
SOFA Baseline	5.28 ± 2.95	4.61 ± 4.17	6.37 ± 2.32	0.145
Horowitz index	142.91 ± 55.91	134.15 ± 60.67	155.31 ± 47.98	0.268
Female at birth%	52.38	53.85	50.00	0.864
Hypertension%	38.10	38.46	37.50	0.965
DM II	23.81	23.08	25.00	0.920
CKF	9.52	0.00	25.00	0.133
Atrial Fibrillation	9.52	0.00	25.00	0.133
Others	38.10%	30.77	50.00	0.378

## Primary endpoint

### PSP

During the study period, we observed increasing plasma PSP levels over time according to mixed model analysis (*p* < 0.001), with higher levels found in the nonsurvivor population (*p* < 0.001). PSP level data were normally distributed at T0, T1, T2, and T3 (*p* value < 0.001, < 0.001, < 0.001, and 0.002, respectively), and significant plasma level differences between survivors and nonsurvivors at each timepoint were observed. PSP measurements achieved a statistically significant result in AUROC analysis with a value higher than 0.7 at T0, T1, T2, and T3 (Table 2). The overall AUROC of PSP was 0.8271 (CI (0.73–0.93), *p* < 0.001), with a sensitivity of 53.13%, a specificity of 92.00%, a positive predictive value of 80.95%, a negative predictive value of 75.41%, and a correct classification percentage of 76.83%.

**Table 2:** PSP plasma levels in COVID-19 ICU patients: it shows differences between survivors and non-survivors for PSP plasma level each time points with Wilcoxon sign rank test, Two-way ANOVA during the time, mixed model coefficients, and Area Under the Receiving Operator sensitivity and specificity for the primary outcome

PSP ng/ml	T0 (21 patients survived)	T1 (21 patients survived)	T2 (21 patients survived)	T3 (21 patients survived)
Survivors (mean ± st. dev)	105.53 ± 28.53	157.76 ± 90.26	102.66 ± 66.94	155.25 ± 119.85
Nonsurvivors (mean ± st. dev)	214.87 ± 134.60	359.75 ± 249.85	399.00 ± 215.15	352.5 ± 119.81
p	0.0098*	0.0148*	0.0003*	0.0048*
			Two-way ANOVA	*P* < 0.001
	PSP	Mortality	Timing	Cons
Correlation coefficients (Rrm)	210.76	204.94	11.93	86.67
P	< 0.001*	< 0.001*	0.042*	0.003*
	T0	T1	T2	T3
AUROC	0.821	0.7019	0.9271	0.9062
*P* value	0.048	0.030	0.036	0.012
Sensitivity	62.50%	50.00%	75.00%	62.50%
Specificity	100.00%	92.31%	91.67%	91.67%
Positive predictive value	100.00%	80.00%	85.71%	83.33%
Negative predictive value	81.25%	75.00%	84.62%	78.57%
Correctly classified	85.71%	76.19%	85.00%	80.00%

### PCT

During the study period, we observed a higher plasma level of PCT in the non-survivor population (*p* = 0.014), but the result in the mixed model analysis over time was not statistically significant. Furthermore, we observed that PCT plasma levels decreased over time. PCT data were not normally distributed at T0, T1, T2, or T3, and significant PCT level differences between survivors and non-survivors were shown only at T0 (*p* = 0.0452). PCT also showed a nonsignificant value in AUROC analyses at T0, T1, T2, and T3 (Table 3). The overall AUROC of PCT was 0.6466 (CI (0.53–0.76), *p* = 0.110), with a sensitivity of 25.81%, a specificity of 95.56%, a positive predictive value of 80.00%, a negative predictive value of 65.15%, and a correct classification percentage of 67.11%.

**Table 3:** PCT plasma levels in COVID-19 ICU patients: it shows differences between survivors and non-survivors for PCT plasma level each time points with Wilcoxon sign rank test, Two-way ANOVA during the time, mixed model coefficients, and Area Under the Receiving Operator sensitivity and specificity for the primary outcome

PCT ng/ml	T0 (21 patients survived)	T1 (21 patients survived)	T2 (21 patients survived)	T3 (21 patients survived)
Survivors (mean ± st. dev)	0.16 ± 0.15	0.40 ± 0.63	0.16 ± 0.12	0.11 ± 0.040
Nonsurvivors (mean ± st. dev)	4.46 ± 9.08	5.67 ± 14.05	1.975 ± 4.49	0.97 ± 1.60
P	0.0452	0.7711	0.2217	0.1950
			Two-way ANOVA	P 0.0176
	PCT	Mortality	Timing	Cons
Correlation coefficient (Rrm)	1.44	3.03	-0.240	1.09
P	0.022*	0.014*	0.299	0.343
	T0	T1	T2	T3
AUROC	0.7396	0.5476	0.6625	0.6307
P value	0.540	0.458	0.352	0.248
Sensitivity	37.50	14.29%	25.00%	37.50
Specificity	100.00%	100.00%	100.00%	100.00%
Positive predictive value	100.00%	100.00%	100.00%	100.00%
Negative predictive value	70.59%	66.67%	62.50%	68.75%
Correctly classified	75%	68.42%	66.67%	73.68%

### CRP

During the study period, a higher plasma level of CRP was observed in the nonsurvivor population, but the difference was not significant. The result in the mixed model analysis over time between survivors and nonsurvivors was not statistically significant. Furthermore, CRP decreased over time. CRP values were not normally distributed at T0, T1, T2, or T3, and no significant differences in plasma levels were detected at T0, T1, T2, or T3. CRP showed a nonsignificant AUROC value at T0, T1, T2, and T3 (Table 4). The overall AUROC of PCT was 0.5816 (CI (0.43–0.70), *p* = 0.178), with a sensitivity of 18.75%, a specificity of 87.76%, a positive predictive value of 50.00%, a negative predictive value of 62.32%, and a correct classification percentage of 60.49%.

**Table 4:** CRP plasma levels in COVID-19 ICU patients: it shows differences between survivors and non-survivors for CRP plasma level each time points with Wilcoxon sign rank test, Two-way ANOVA during the time, mixed model coefficients, and Area Under the Receiving Operator sensitivity and specificity for the primary outcome

CRP mg/dl	T0 (21 patients survived)	T1 (21 patients survived)	T2 (21 patients survived)	T3 (21 patients survived)
Survivors (mean ± st. dev)	9.88 ± 7.34	5.01 ± 6.49	2.01 ± 3.16	1.60 ± 2.53
Nonsurvivors (mean ± st. dev)	8.37 ± 8.04	8.05 ± 9.26	4.98 ± 5.57	6.03 ± 7.74
P	0.5817	0.6075	0.1738	0.2177
			Two-way ANOVA	P 0.1394
	CRP	Mortality	Timing	Cons
Correlation coefficient (Rrm)	5.60	2.15	-0.92	8.18
P	< 0.001*	0.127	< 0.001*	< 0.001*
	T0	T1	T2	T3
AUROC	0.5769	0.5729	0.6823	0.6615
*P* value	0.645	0.394	0.151	0.119
Sensitivity	0.00%	25.00	37.50	37.50
Specificity	100.00%	83.33%	83.33%	91.67%
Positive predictive value	0.00%	50.00%	60.00%	75.00%
Negative predictive value	61.90%	62.50%	66.67%	68.75%
Correctly classified	61.90%	60.00%	65.00%	70.00%

**Fig. 2:**
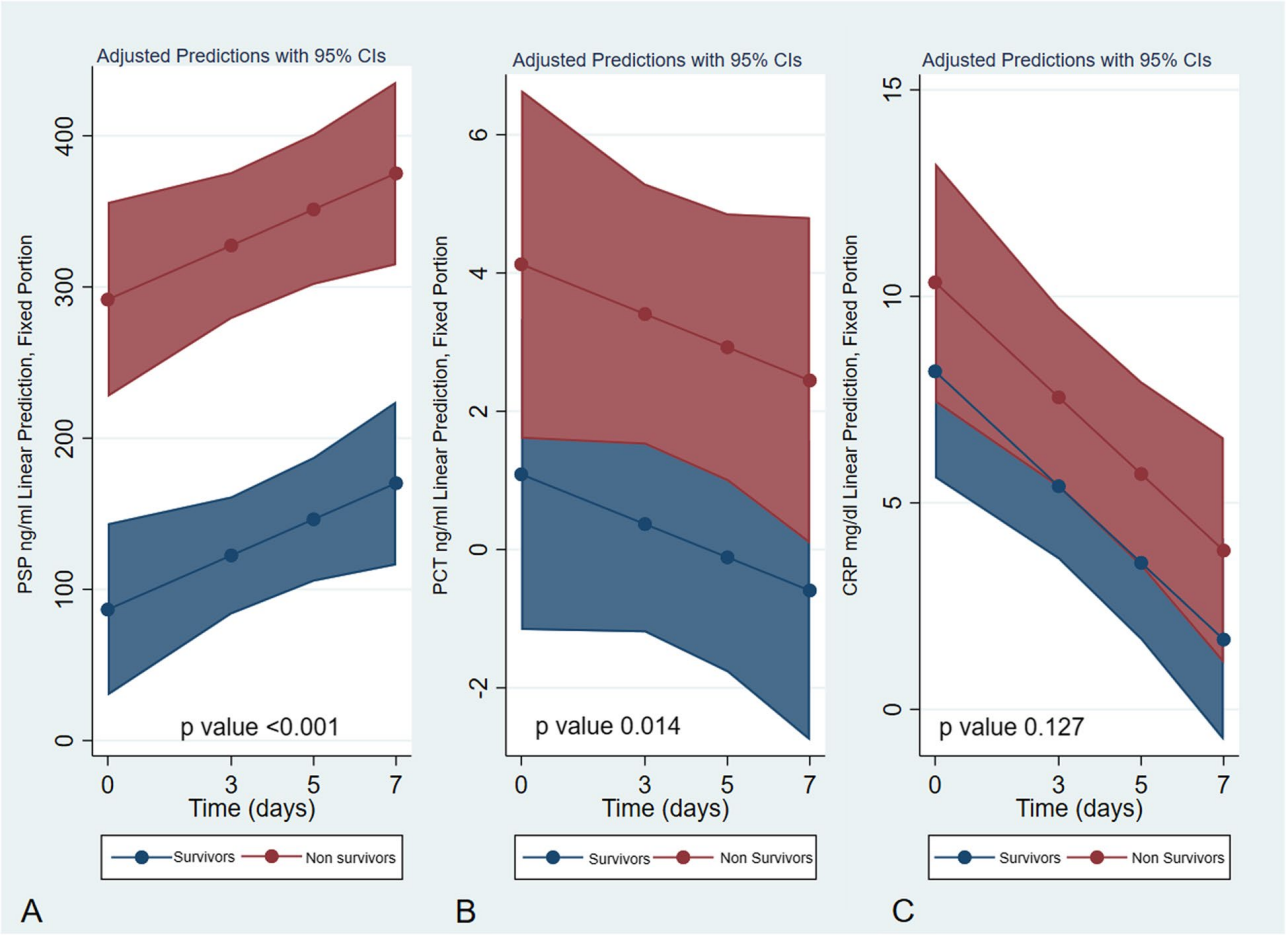
Shows the postestimation margin plot of the repeated measures mixed model between survivors and non-survivors for PSP (panel **A**), PCT (panel **B**), and CRP (panel **C**)

**Fig. 3:**
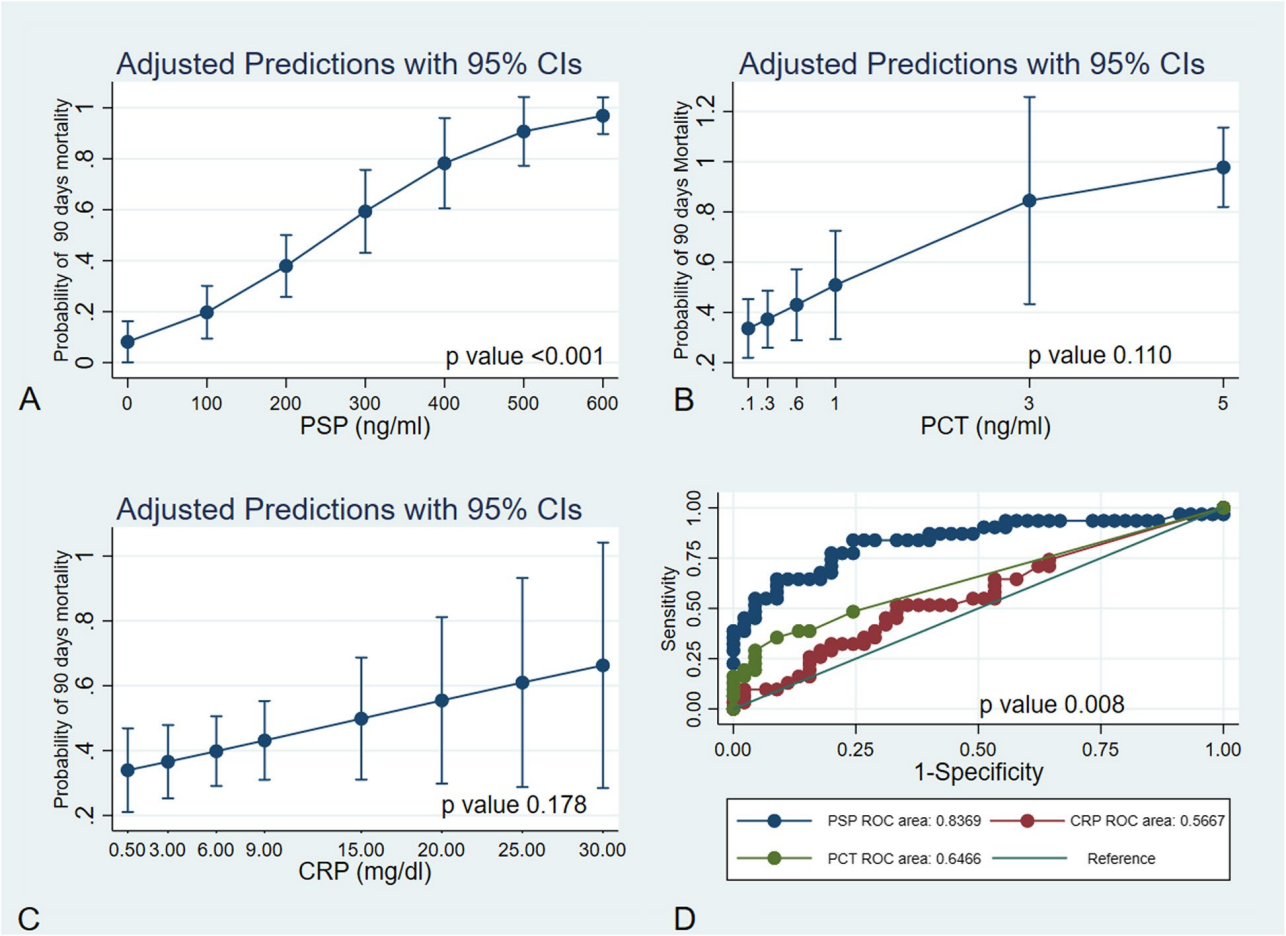
Shows the postestimation margin plots, with the probability of death plotted on the y-axis and the level of PSP (panel **A**), PCT (panel **B**) and CRP (panel **C**) plotted on the x-axis. Panel D shows the overall AUROC comparison for PSP, PCT and CRP

As shown in Fig. 2 (Fig. 2: panel A-B-C), the difference between survivors and nonsurvivors observed in the mixed model analysis was significant for PSP both for mortality and timing. However, the results for PCT and CRP were not significant for either mortality or timing. Figure 2 displays the postestimation probability of death and the levels of PSP, PCT, and CRP (Fig. 3 panel A-B-C). The overall AUROC comparison shows a higher significant value for PSP *than for* PCT and CRP (Fig. 3 panel D and Table 5).

**Table 5:** AUROC comparison among PSP, PCT and CRP plasma levels in COVID-19 ICU patients

	AUROC	CI	P value	Bonferroni *p* value
*PSP*	0.8360	0.73540 0.93664		
*PCT*	0.6471	0.53348 0.76068	0.002*	< 0.001*
*CRP*	0.5718	0.43890 0.70473	< 0.001*	< 0.001*

## Discussion

We investigated the PSP levels in critically ill COVID-19 patients, and we found the PSP level to be a helpful marker in evaluating the severity of illness and in predicting mortality over 90 days. PSP is secreted in the pancreas, small intestine and stomach and has been shown to be upregulated in the presence of infection and organ dysfunction; in fact, PSP plasma levels are significantly increased [4, 17, 18]. PSP may have an activating effect on leukocytes, triggering polymorphonuclear neutrophil granulocyte activation. This activation facilitates the adherence of selectins and integrins on the neutrophil surface to the vessel walls, i.e., to the endothelial cells; this mechanism plays an essential role in the development of pulmonary oedema and acute respiratory distress syndrome (ARDS) observed in severe COVID-19 infections [19, 20]. The loss of endothelial integrity is crucial in COVID-19 [21, 22]. This phenomenon could explain why PSP plasma levels increase over time in critically ill COVID-19 patients. It is important to underline that PSP is not an inflammatory protein but a direct indicator of an inflammatory state. In this study, PSP performed better than PCT and CRP as a biomarker for disease progression, in agreement with Llewelyn et al. [23]. Plasma biomarkers such as CRP and PCT are insufficiently sensitive or specific in predicting the onset of severe septic shock, ARDS and COVID-19-related acute viral septic shock in these critically ill patients [17, 24, 25]. Several studies have evaluated the role of CRP in COVID-19. Some of them found that elevated CRP on admission for patients with COVID-19 was associated with increased in-patient mortality and was indicative of disease severity at admission [26–28], while other studies documented no significant differences in the CRP level among mild, severe, and critical patients. The role of CRP remains controversial in the medical literature about its prognostic role: according to Pierrakos et al., quantification of this protein is widely used, but it has limited ability to distinguish sepsis from other inflammatory conditions or to predict the outcome [29, 30]. PSP has the potential to perform better, and this is the first study documenting the potential role of PSP for risk stratification in COVID-19 patients. Additionally, the availability of this test at the point-of-care may provide further advantages through faster results and timely clinical decisions.

PSP showed an AUROC value higher than 0.7 at each measurement timepoint, as well as a significant result in the mixed model analysis over time and a significant difference in plasma levels between survivors and non-survivors; these differences were not observed for PCT and CRP plasma levels. Multiple studies showed that PCT levels show no significant increase in COVID-19 patients and the presented results for PCT are not surprising [3, 31]. Therefore, increased PCT levels may be a useful marker to detect the emergence of secondary bacterial infection in critical care patients [32]. PCT showed a lower sensitivity compared to PSP and these results partially agree with results from Que et al. and Gukasjan et al. PCT probably has better sensitivity in case of septic shock due bacterial infection and severity of sepsis [17, 33]. Specifically, PCT levels are important markers of disease severity in case of sepsis, as shown be Que et al. [34] among others.

Additionally, while bacterial sepsis and COVID-19 are two distinct disease entities, each underpinned by its pathophysiology, the two frequently overlap as sepsis is a common late complication of COVID-19 in the ICU population, partly due to direct effects of the primary viral infection on the immune system, but also due to iatrogenic complications of immunomodulatory medications, invasive ventilation as well as other invasive procedure that are commonly performed in this setting. There are several differences between the two, as in the bacterial sepsis the systemic inflammation leads an early and sudden clinical deterioration, while COVID-19 has demonstrated a complex pathogenesis that involves other mechanisms of tissue damage and typically a late clinical deterioration in the disease course [35]. According to Fidalgo et al. PSP accuracy for the diagnosis of infection and sepsis seems to be at least comparable to the biomarkers currently used in clinical practice. Furthermore, it seems to outperform those biomarkers in the prediction of sepsis, because PSP levels seem to anticipate the clinical diagnosis [36–38]. PSP also seems to have a good prognostic value for mortality at 28 days also in sepsis especially if combined with blood lactate or PCT [5, 34]. We hypothesize that PSP may have better prognostic performance compared to other biomarkers because it is affected both by the primary viral disease and by sepsis, which is a common complication in the late course of COVID-19. This may be particularly useful for prognostication in the late course of the disease. Other studies that have compared biomarkers for prognosis in COVID-19 have used samples from the time of admission, emergency department presentation or unspecified time and have not found significant differences in performance with PCT or CRP, while ours has used samples collected at multiple times, including in the late phase of disease [2, 8]. Lastly, the use of this biomarker does not exclude the use of others, as certainly integration of data may provide important and complementary information.

The cytokine storm in COVID-19 suggested that IL-6 might be useful as prognostic biomarker, hence medical research investigated this question [39–41]. Gorham et al. demonstrated the value of repeated measurements of IL-6 in critically severe COVID-19 patients, identifying patients with a high risk of poor prognosis. During the COVID-19 pandemic, IL-6 plasma levels progressively became a routine laboratory exam at our institution. However, due to the high demand and limited capacity during the study period, the serial measurement of plasma IL-6 cytokines was not possible. Consequently, we were not able to compare PSP and IL-6, which has been shown in several studies to be associated with the degree of disease severity [42–45]. In a study by Que et. al that compared PSP and IL-6 in predicting mortality, PSP performed better than IL-6. While IL-6 appears to be an accurate prognostic marker, the measured plasma levels do not seem to perform equally good as treatment response markers [17]. Unfortunately, due to the availability issues at our institution of IL-6 testing, we could not compare IL-6 and PSP as treatment response biomarkers. Another possible biomarker to predict evolution in COVID-19 patients is D-dimer. In some studies, coagulation measured D-dimer levels were significantly higher in patients who developed ARDS and died than in patients who survived [46–48]. However, the interpretation of D-dimer during disease monitoring is currently unclear, as it may not be directly related to disease severity [49].

The absence of specific prognostic biomarkers in severe COVID-19 makes the research on the role of PSP through point-of-care technology even more interesting. Observing the post-estimation model (Fig. 3 A), it is possible to infer that a value of PSP, at the time of ICU admission, between 250–300 ng/ml is associated with a probability of death at 90 days above 50% (Fig. 3 panel A). However, while our study has multiple measures for every patient by design, the sample size for PSP levels at admission is not enough to clearly answer this research question. Studies with larger sample size are granted to clearly identify and validate a cut-off of PSP blood levels ad a prognostic biomarker. Recently, medical researchers investigated the role of various endothelial proteins in COVID-19, and some researchers have measured PSP using ELISA methods [50, 51]. In contrast, point-of-care technology provides a quick result (PSP results are available within 7.5 min with the abioSCOPE platform), which allows physicians to triage patients according to the severity of illness and to start the most appropriate medical treatment as soon as possible, in conformity with the “golden hour”, although a little more expensively than the ELISA method. The advantages of this technology are already widely known, as it is used in coagulation and thromboelastography tests. This study was carried out following the CONSORT guidelines. This research has limitations, mainly in the single-center nature of the study and in the small sample of patients enrolled. As a consequence, our results do not allow for generalization to other settings. We find the prospective observational nature with repeated measures a strength of our study.

## Conclusions

These first results suggest the potential advantages of monitoring PSP levels in predicting long-term mortality with the help of a point-of-care technology, which can provide results in under 10 min and at the patient’s bedside. Furthermore, our results showed a low sensitivity of PCT compared to PSP, so we conclude that monitoring and measuring the clinical course of this protein may be helpful. Monitoring PSP levels with point-of-care technology could prove to be useful in the absence of a specific and clinically validated COVID-19 biomarker. Additional data are needed to confirm these findings.

## Fundings

We received PSP kit measurement generously to conduct the study from Aferetica S.r.l, San Giovanni Persiceto, (Bologna), Italy.

## Data Availability

Data are available upon request to the authors. Point of contacts: melegari.gabriele@gmail.com.

## References

[1] Boeck L, Graf R, Eggimann P, Pargger H, Raptis DA, Smyrnios N, Thakkar N, Siegemund M, Rakic J, Tamm M (2011). Pancreatic stone protein: a marker of organ failure and outcome in ventilator-associated pneumonia. Chest.

[2] Van Singer M, Brahier T, Brochu Vez MJ, Gerhard Donnet H, Hugli O, Boillat-Blanco N (2021). Pancreatic stone protein for early mortality prediction in COVID-19 patients. Crit Care.

[3] Bivona G, Agnello L, Ciaccio M. Biomarkers for prognosis and treatment response in COVID-19 Patients. Ann Lab Med. 2021;41(6):540–8. 10.3343/alm.2021.41.6.540 PMC8203437 34108281

[4] Izcovich A, Ragusa MA, Tortosa F, Lavena Marzio MA, Agnoletti C, Bengolea A, Ceirano A, Espinosa F, Saavedra E, Sanguine V, et al. Prognostic factors for severity and mortality in patients infected with COVID-19: a systematic review. PLoS One. 2020;15(11). 10.1371/journal.pone.0241955 PMC7671522 33201896

[5] García de Guadiana-Romualdo L, Albaladejo-Otón MD, Berger M, Jiménez-Santos E, Jiménez-Sánchez R, Esteban-Torrella P, Rebollo-Acebes S, Hernando-Holgado A, Ortín-Freire A, Trujillo-Santos J: Prognostic performance of pancreatic stone protein in critically ill patients with sepsis. Biomark Med 2019, 13(17):1469–1480. 10.2217/bmm-2019-0174 31621373

[6] Klein HJ, Niggemann P, Buehler PK, Lehner F, Schweizer R, Rittirsch D, Fuchs N, Waldner M, Steiger P, Giovanoli P (2021). Pancreatic stone protein predicts sepsis in severely burned patients irrespective of trauma severity: a monocentric observational study. Ann Surg.

[7] Pugin J, Daix T, Pagani JL, Morri D, Giacomucci A, Dequin PF, Guitton C, Que YA, Zani G, Brealey D (2021). Serial measurement of pancreatic stone protein for the early detection of sepsis in intensive care unit patients: a prospective multicentric study. Crit Care.

[8] Lagadinou M, Paraskevas T, Velissaris D, Michailides C, Eleftherakis G, Sampsonas F, Siakallis G, Assimakopoulos SF, Marangos M (2022). The role of pancreatic stone protein as a prognostic factor for COVID-19 patients. Eur Rev Med Pharmacol Sci.

[9] Pandian V, Morris LL, Brodsky MB, Lynch J, Walsh B, Rushton C, Phillips J, Rahman A, DeRose T, Lambe L, et al. Critical care guidance for tracheostomy care during the COVID-19 Pandemic: A Global, Multidisciplinary Approach. Am J Crit Care. 2020;29(6):e116–27. 10.4037/ajcc2020561 32929453

[10] Cook TM, El-Boghdadly K, McGuire B, McNarry AF, Patel A, Higgs A (2020). Consensus guidelines for managing the airway in patients with COVID-19: guidelines from the difficult airway society, the association of anaesthetists the intensive care society, the faculty of intensive care medicine and the royal college of anaesthetists. Anaesthesia.

[11] Cuker A, Tseng EK, Nieuwlaat R, Angchaisuksiri P, Blair C, Dane K, Davila J, DeSancho MT, Diuguid D, Griffin DO (2021). American society of hematology 2021 guidelines on the use of anticoagulation for thromboprophylaxis in patients with COVID-19. Blood Adv.

[12] King TS, Chinchilli VM, Wang KL, Carrasco JL (2007). A class of repeated measures concordance correlation coefficients. J Biopharm Stat.

[13] Shan G, Zhang H, Jiang T (2020). Correlation coefficients for a study with repeated measures. Comput Math Methods Med.

[14] Cohen J (1992). A power primer. Psychol Bull.

[15] Faul F, Erdfelder E, Buchner A, Lang AG: Statistical power analyses using G*Power 3.1: tests for correlation and regression analyses. Behav Res Methods 2009, 41(4):1149–1160. 10.3758/BRM.41.4.1149 19897823

[16] Faul F, Erdfelder E, Lang AG, Buchner A (2007). G*Power 3: a flexible statistical power analysis program for the social, behavioral, and biomedical sciences. Behav Res Methods.

[17] Que YA, Delodder F, Guessous I, Graf R, Bain M, Calandra T, Liaudet L, Eggimann P (2012). Pancreatic stone protein as an early biomarker predicting mortality in a prospective cohort of patients with sepsis requiring ICU management. Crit Care.

[18] Lopes D, Chumbinho B, Bandovas JP, Faria P, Espírito Santo C, Ferreira B, Val-Flores L, Pereira R, Germano N, Bento L (2022). Pancreatic stone protein as a biomarker of sepsis. Crit Care.

[19] Peterson MW, Stone P, Shasby DM: Cationic neutrophil proteins increase transendothelial albumin movement. J Appl Physiol (1985) 1987, 62(4):1521–1530. 10.1152/jappl.1987.62.4.1521 3648023

[20] Keel M, Härter L, Reding T, Sun LK, Hersberger M, Seifert B, Bimmler D, Graf R (2009). Pancreatic stone protein is highly increased during posttraumatic sepsis and activates neutrophil granulocytes. Crit Care Med.

[21] Price DR, Benedetti E, Hoffman KL, Gomez-Escobar L, Alvarez-Mulett S, Capili A, Sarwath H, Parkhurst CN, Lafond E, Weidman K (2022). Angiopoietin 2 is associated with vascular necroptosis induction in coronavirus disease 2019 acute respiratory distress syndrome. Am J Pathol.

[22] Villa E, Critelli R, Lasagni S, Melegari A, Curatolo A, Celsa C, Romagnoli D, Melegari G, Pivetti A, Di Marco L (2021). Dynamic angiopoietin-2 assessment predicts survival and chronic course in hospitalized patients with COVID-19. Blood Adv.

[23] Llewelyn MJ, Berger M, Gregory M, Ramaiah R, Taylor AL, Curdt I, Lajaunias F, Graf R, Blincko SJ, Drage S (2013). Sepsis biomarkers in unselected patients on admission to intensive or high-dependency care. Crit Care.

[24] Evans L, Rhodes A, Alhazzani W, Antonelli M, Coopersmith CM, French C, Machado FR, McIntyre L, Ostermann M, Prescott HC (2021). Surviving sepsis campaign: international guidelines for management of sepsis and septic shock 2021. Intensive Care Med.

[25] Parasher A (2021). COVID-19: Current understanding of its Pathophysiology, Clinical presentation and Treatment. Postgrad Med J.

[26] Stringer D, Braude P, Myint PK, Evans L, Collins JT, Verduri A, Quinn TJ, Vilches-Moraga A, Stechman MJ, Pearce L (2021). The role of C-reactive protein as a prognostic marker in COVID-19. Int J Epidemiol.

[27] Nori W (2022). C-Reactive protein role in assessing COVID-19 deceased geriatrics and survivors of severe and critical illness. World J Clin Cases.

[28] Acar E, Demir A, Yıldırım B, Kaya MG, Gökçek K (2021). The role of hemogram parameters and C-reactive protein in predicting mortality in COVID-19 infection. Int J Clin Pract.

[29] Pierrakos C, Vincent JL (2010). Sepsis biomarkers: a review. Crit Care.

[30] Luo W, Zhang JW, Zhang W, Lin YL, Wang Q (2021). Circulating levels of IL-2, IL-4, TNF-α, IFN-γ, and C-reactive protein are not associated with severity of COVID-19 symptoms. J Med Virol.

[31] Malik P, Patel U, Mehta D, Patel N, Kelkar R, Akrmah M, Gabrilove JL, Sacks H (2021). Biomarkers and outcomes of COVID-19 hospitalisations: systematic review and meta-analysis. BMJ Evid Based Med.

[32] Pink I, Raupach D, Fuge J, Vonberg RP, Hoeper MM, Welte T, Rademacher J (2021). C-reactive protein and procalcitonin for antimicrobial stewardship in COVID-19. Infection.

[33] Gukasjan R, Raptis DA, Schulz HU, Halangk W, Graf R (2013). Pancreatic stone protein predicts outcome in patients with peritonitis in the ICU. Crit Care Med.

[34] Que YA, Guessous I, Dupuis-Lozeron E, de Oliveira CRA, Oliveira CF, Graf R, Seematter G, Revelly JP, Pagani JL, Liaudet L (2015). Prognostication of mortality in critically III patients with severe infections. Chest.

[35] Koçak Tufan Z, Kayaaslan B, Mer M: COVID-19 and Sepsis. Turk J Med Sci. 2021; 51(Si-1):3301–3311. 10.3906/sag-2108-239 PMC8771020 34590796

[36] Fidalgo P, Nora D, Coelho L, Povoa P: Pancreatic stone protein: review of a new biomarker in Sepsis. J Clin Med. 2022, 11(4):1085. 10.3390/jcm11041085 PMC8880320 35207355

[37] Ventura F, Gasche Y, Rached AKB, Pugin D, Mollard F, Vora S, Charbonnet P, Bühler L: Pancreatic stone protein as a biomarker for the early diagnosis of post-operative peritonitis, intra-abdominal infection and sepsis. J Surg Case Rep 2022, 2022(11):rjac497. 10.1093/jscr/rjac497 PMC9659425 36389436

[38] Póvoa P, Martin-Loeches I, Ramirez P, Bos LD, Esperatti M, Silvestre J, Gili G, Goma G, Berlanga E, Espasa M (2016). Biomarker kinetics in the prediction of VAP diagnosis: results from the BioVAP study. Ann Intensive Care.

[39] Galván-Román JM, Rodríguez-García SC, Roy-Vallejo E, Marcos-Jiménez A, Sánchez-Alonso S, Fernández-Díaz C, Alcaraz-Serna A, Mateu-Albero T, Rodríguez-Cortes P, Sánchez-Cerrillo I (2021). IL-6 serum levels predict severity and response to tocilizumab in COVID-19: an observational study. J Allergy Clin Immunol.

[40] Han H, Ma Q, Li C, Liu R, Zhao L, Wang W, Zhang P, Liu X, Gao G, Liu F (2020). Profiling serum cytokines in COVID-19 patients reveals IL-6 and IL-10 are disease severity predictors. Emerg Microbes Infect.

[41] Jøntvedt Jørgensen M, Holter JC, Christensen EE, Schjalm C, Tonby K, Pischke SE, Jenum S, Skeie LG, Nur S, Lind A (2020). Increased interleukin-6 and macrophage chemoattractant protein-1 are associated with respiratory failure in COVID-19. Sci Rep.

[42] Coomes EA, Haghbayan H (2020). Interleukin-6 in Covid-19: a systematic review and meta-analysis. Rev Med Virol.

[43] Santa Cruz A, Mendes-Frias A, Oliveira AI, Dias L, Matos AR, Carvalho A, Capela C, Pedrosa J, Castro AG, Silvestre R (2021). Interleukin-6 is a biomarker for the development of fatal severe acute respiratory syndrome coronavirus 2 pneumonia. Front Immunol.

[44] Schultheiß C, Willscher E, Paschold L, Gottschick C, Klee B, Henkes SS, Bosurgi L, Dutzmann J, Sedding D, Frese T (2022). The IL-1β, IL-6, and TNF cytokine triad is associated with post-acute sequelae of COVID-19. Cell Rep Med.

[45] Van Singer M, Brahier T, Ngai M, Wright J, Weckman AM, Erice C, Meuwly JY, Hugli O, Kain KC, Boillat-Blanco N (2021). COVID-19 risk stratification algorithms based on sTREM-1 and IL-6 in emergency department. J Allergy Clin Immunol.

[46] Lippi G, Favaloro EJ (2020). D-dimer is associated with severity of coronavirus disease 2019: a pooled analysis. Thromb Haemost.

[47] Iba T, Levy JH, Levi M, Thachil J (2020). Coagulopathy in COVID-19. J Thromb Haemost.

[48] Li Y, Zhao K, Wei H, Chen W, Wang W, Jia L, Liu Q, Zhang J, Shan T, Peng Z (2020). Dynamic relationship between D-dimer and COVID-19 severity. Br J Haematol.

[49] Ponti G, Maccaferri M, Ruini C, Tomasi A, Ozben T (2020). Biomarkers associated with COVID-19 disease progression. Crit Rev Clin Lab Sci.

[50] Bouck EG, Denorme F, Holle LA, Middelton EA, Blair AM, de Laat B, Schiffman JD, Yost CC, Rondina MT, Wolberg AS (2021). COVID-19 and Sepsis are associated with different abnormalities in plasma procoagulant and fibrinolytic activity. Arterioscler Thromb Vasc Biol.

[51] Haffke M, Freitag H, Rudolf G, Seifert M, Doehner W, Scherbakov N, Hanitsch L, Wittke K, Bauer S, Konietschke F (2022). Endothelial dysfunction and altered endothelial biomarkers in patients with post-COVID-19 syndrome and chronic fatigue syndrome (ME/CFS). J Transl Med.

